# Default Mode, Dorsal Attention and Auditory Resting State Networks Exhibit Differential Functional Connectivity in Tinnitus and Hearing Loss

**DOI:** 10.1371/journal.pone.0076488

**Published:** 2013-10-02

**Authors:** Sara A. Schmidt, Kwaku Akrofi, Jake R. Carpenter-Thompson, Fatima T. Husain

**Affiliations:** 1 Neuroscience Program, University of Illinois Urbana-Champaign, Champaign, Illinois, United States of America; 2 Department of Speech and Hearing Science, University of Illinois Urbana-Champaign, Champaign, Illinois, United States of America; 3 Beckman Institute for Advanced Science and Technology, University of Illinois Urbana-Champaign, Champaign, Illinois, United States of America; University of Michigan, United States of America

## Abstract

We investigated auditory, dorsal attention, and default mode networks in adults with tinnitus and hearing loss in a resting state functional connectivity study. Data were obtained using continuous functional magnetic resonance imaging (fMRI) while the participants were at “rest” and were not performing any task. Participants belonged to one of three groups: middle-aged adults with tinnitus and mild-to-moderate high frequency hearing loss (TIN), age-matched controls with normal hearing and no tinnitus (NH), and a second control group with mild-to-moderate high frequency hearing loss without tinnitus (HL). After standard preprocessing, (a) a group independent component analysis (ICA) using 30 components and (b) a seeding-based connectivity analysis were conducted. In the group ICA, the default mode network was the only network to display visual differences between subject groups. In the seeding analysis, we found increased connectivity between the left parahippocampus and the auditory resting state network in the TIN group when compared to NH controls. Similarly, there was also an increased correlation between the right parahippocampus and the dorsal attention network when compared to HL controls. Other group differences in this attention network included decreased correlations between the seed regions and the right supramarginal gyrus in TIN patients when compared to HL controls. In the default mode network, there was a strong decrease in correlation between the seed regions and the precuneus when compared to both control groups. The findings of this study identify specific alterations in the connectivity of the default mode, dorsal attention, and auditory resting state networks due to tinnitus. The results suggest that therapies for tinnitus that mitigate the increased connectivity of limbic regions with auditory and attention resting state networks and the decreased coherence of the default mode network could be effective at reducing tinnitus-related distress.

## Introduction

Spontaneous fluctuations in the blood oxygenation level-dependent (BOLD) response have been shown to reliably organize into spatially independent networks [1–5]. One of the first such networks to be observed was the ‘default mode network’ (DMN) [5]. The DMN is found to be particularly active during rest, when no task is being performed. The regions of this network include the medial prefrontal cortex, posterior cingulate cortex, precuneus, bilateral superior frontal gyri, and bilateral inferior parietal lobules [4]. In a task-based scenario, the network deactivates [5]. Many other resting state networks have since been identified [4,6]. The non-invasive, relatively simple data collection associated with the resting state has made it an appealing way to study differences in connectivity between healthy controls and various populations with neurological disorders, including Alzheimer’s disease [7–12], schizophrenia [13–18], depression [19], and post-traumatic stress disorder [20].

Recently, the analysis of resting state functional connectivity has been applied to patients with tinnitus. Tinnitus, defined as the perception of sound in the absence of an external stimulus, affects up to 50 million people in the United States according to the American Tinnitus Association. Of these, 16 million people are bothered enough to seek medical help for their tinnitus, and 2 million are severely debilitated by the disorder [21]. Treatment options for tinnitus currently focus on building habituation to the stimulus, thus enabling a patient to ignore the sound they are experiencing and live with it without any problem. Treatments to eliminate the phantom sound are as yet non-existent, due in part to the unclear nature of the neurologic underpinnings of tinnitus. Task-based functional imaging studies have begun to untangle the influence tinnitus has on neural networks, revealing that tinnitus has an impact on cognition, attention and emotional processing networks in particular [22]. However, there are few resting state functional connectivity studies of tinnitus [23–27]. Resting state analysis offers several advantages over task-based fMRI studies. Differences between tinnitus patients and normal controls found in task-based studies could be influenced by changes in baseline activity [22]. The use of resting state fMRI (rs-fMRI) complements task-based data well by exploring this baseline. It also eliminates concerns brought about by tinnitus and hearing loss, such as whether or not subjects can hear presented stimuli [23]. Most importantly, however, it explores changes in neural activity that occur when patients are most bothered by their tinnitus: at rest, such as when they are attempting to fall asleep. Our study examined three resting state networks likely to be influenced by tinnitus: the auditory resting state network, as tinnitus is an auditory phenomenon; the default mode network, which exhibits the most coherent activity at rest; and the dorsal attention network, as tinnitus patients often attend to their phantom percept. This attention network was selected because it has been shown to be strongly anti-correlated with the default mode network [2]; other attention-related resting state networks were not examined in this study.

Recent publications concerning tinnitus and rs-fMRI provide inconsistent results. The first preliminary study [24] found a link between the auditory resting state network (RSN) and the limbic system, specifically the amygdala, in four patients with tinnitus when compared to six normal hearing controls. In that study, a seed-based analysis was used; regions of interest were chosen based on the results of a group independent component analysis (group ICA). A separate study [26] employing group ICA and graph theory developed connectivity maps demonstrating the increased connectivity between auditory and limbic regions, including the left parahippocampal region, in tinnitus patients. In this study, which used a larger cohort of 13 tinnitus patients with varying severity, connectivity changes were found in limbic and parahippocampal areas, as well as in basal ganglia, higher-order prefrontal and parietal associative networks, the brainstem, the cerebellum, and sensory-motor and visual-motor systems. A companion study [25] using graph connectivity analysis found two different connectivity patterns in the auditory resting state network. The first network, consisting of bilateral auditory cortices and the insula, was found in both tinnitus patients and normal hearing controls. The second network included the frontoparietal lobe, the anterior cingulate cortex, the amygdala, the brainstem, and the parahippocampus. This network was anticorrelated with the time course of the auditory network and was found only in control subjects.

All of these studies, however, made no attempt to control for tinnitus severity. In the Maudoux et al. papers [25,26], THI scores ranged from 16 to 84. In the Kim et al. paper [24], no mention of tinnitus severity was made. Additional studies have recently been published focusing separately on bothersome and non-bothersome tinnitus [23,27]. It is important to study both groups of individuals to fully comprehend the disorder; non-bothersome tinnitus is by far the more common situation, but those with bothersome tinnitus are more in need of treatment. In bothersome tinnitus, use of a seed-based analysis revealed positive correlations between auditory and visual regions in the control group, but negative correlations between those areas in the tinnitus group. No significant differences between groups were found in areas in the dorsal attention network (DAN) or the DMN. However, areas involved in the executive control of attention, including the right middle and inferior frontal gyri and the right anterior insula, were found to be positively correlated with the auditory cortex and negatively correlated with parts of the occipital cortex in tinnitus patients. All of these changes could be attributed to coping with the distraction of a phantom sound. Visual networks may be deactivated because they are not necessary in attending to the auditory stimulus; changes in attention networks may be present to decrease the attention paid to the phantom stimulus and allow attention to be given to non-auditory stimuli [23]. In the non-bothersome tinnitus study, the same method of analysis revealed no significant changes in resting state connectivity as compared to controls. It is possible that changes to resting state networks are brought about as a response to the bothersome or stressful nature of the disorder as opposed to the phantom stimulus itself [27].

Though the latter two studies control for tinnitus severity [23,27], they make no attempt to control for hearing loss. Hearing loss and tinnitus often occur together; 90% of those with tinnitus also have some form of hearing loss, but only 30-40% of those with hearing loss also develop tinnitus [28,29]. Hearing loss may also have an impact on resting state functional connectivity, and it is important to dissociate the effect of tinnitus from that due to hearing loss alone when evaluating existing therapies or developing new ones. Including a hearing loss group to serve as a second control helps to identify and separate the effects of hearing loss and tinnitus on connectivity. This approach will tell us nothing about normal hearing patients coping with tinnitus, but it gives us information about the great majority of the tinnitus population (those with both tinnitus and hearing loss) [22].

In our study, three groups of participants were used: a non-bothersome tinnitus group with hearing loss, a control group with normal hearing, and a second control group with matching hearing loss without tinnitus. The auditory RSN, DMN, and DAN were examined using separate group ICA and seeding analyses. We hypothesized that tinnitus, more so than hearing loss, would affect resting state functional connectivity. Sensory deprivation (specifically, the reduction in auditory input) has been shown to elevate activity in auditory regions [30–32]. However, elevated activity would not necessarily be reflected in changes in resting state connectivity, resulting in similar correlation patterns in the two control groups. We hypothesized that altered response patterns found in tinnitus patients in limbic [33,34], somatosensory [35], and attention regions [22,30] would be echoed in the auditory RSN. We expected the DAN to show decreased coherence in TIN subjects compared to either of the control groups because the act of attending or suppressing the tinnitus sound would cause a differential response pattern across network nodes. We further hypothesized a decreased connectivity pattern of the DMN relative to the control groups because the network is known to deactivate when sensory stimuli are processed [5], as is likely the case when processing the internal tinnitus sound.

## Methods

### Ethics Statement

Approval for this study was obtained from the University of Illinois at Urbana-Champaign Institutional Review Board. Participants were recruited from the Urbana-Champaign area and were scanned under the UIUC IRB 10144 protocol. Written informed consent was obtained from each participant and they were suitably compensated.

### Participants

**Figure 1:**
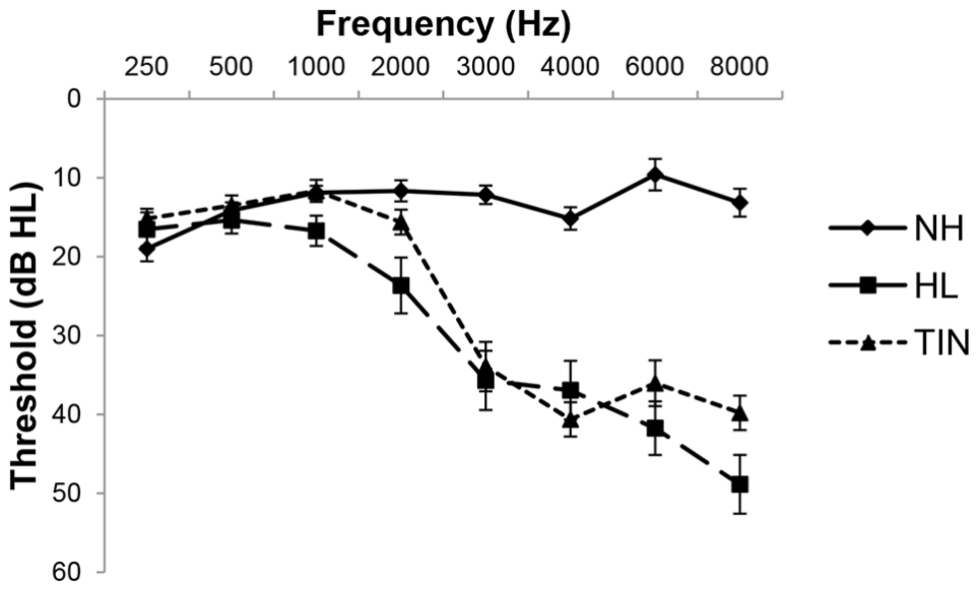
Average audiograms of the participants in the three groups. The error bars represent the standard error of the mean. Thresholds from the left and right ears were averaged for each participant prior to being entered in the group mean.

Three groups of participants were examined. The first group (TIN) contained twelve patients (mean age 55.00±6.97, 3 female) with chronic, non-bothersome tinnitus. All TIN subjects had either "slight" or "mild" tinnitus; the mean Tinnitus Handicap Inventory (THI) score was 8.33±6.76, with a high score of 18 and a low score of 0. None had hyperacusis, as measured by a brief in-house questionnaire and loudness discomfort levels. The second group (HL) contained thirteen age-matched patients (mean age 57.62±9.39, 8 female) with hearing loss but no tinnitus. Hearing loss in both the HL and TIN groups was minimal from 250-2000 Hz and moderate to moderately-severe (threshold ≥ 35 dB) for 3-8 kHz (see Figure 1 for average audiogram). A third age-matched control group of fifteen participants (NH) (mean age 52.93±8.64, 6 female) with normal hearing at all testing frequencies (0.25, 0.5, 1, 2, 4, 6, and 8 kHz) was also included. See Table 1 for a summary of demographic information.

**Table 1:** Subject demographics and clinical characteristics for each subject group.

	**Normal Hearing**	**Hearing Loss**	**Tinnitus**
**Group Size**	15	13	12
**Age**	52.93±8.64	57.62±9.39	55.00±6.97
**Gender**	6 F/9 M	8 F/5 M	3 F/9 M
**Handedness**	14 R/1 L	12 R/1 L	11 R/1 L
**BAI (mean±standard deviation)**	1.13±1.30	2.31±3.36	0.75±1.06
**BDI-II (mean±standard deviation)**	1.67±2.47	4.53±4.82	1.25±1.96
**THI (mean±standard deviation)**	N/A	N/A	8.33±6.76

BDI: Beck Depression Inventory; BAI: Beck Anxiety Inventory; THI: Tinnitus Handicap Inventory.

### Image Acquisition

Magnetic resonance images were taken using a 3T Siemens Magnetom Allegra MRI head-only scanner. BOLD contrast responses were collected for each subject using a gradient echo-planar (EPI) sequence with axial orientation (repetition time [TR] = 2000 ms, echo time [TE] = 30 ms, flip angle = 90°, 32 slices, voxel size = 3.4 × 3.4 × 4.0 mm^3^). Two structural images for each subject were also captured: a high-resolution, T1-weighted, sagittal MPRAGE image (TR=2300ms, TE=2.83 ms, flip angle = 9°, 160 slices, voxel size = 1.0 ×1.0 × 1.2 mm^3^), and a lower-resolution, T2-weighted, axial TSE image (TR=7260 ms, TE=98 ms, flip angle = 150°, 32 slices, voxel size = 0.9 × 0.9 × 4.0 mm^3^).

During the functional scan, subjects were instructed to lay still and look at a fixation cross for the scan duration, which was continuous and lasted approximately five minutes. 150 volumes were collected for each subject. The first four images were discarded prior to preprocessing, leaving 146 volumes for analysis. The tinnitus of four of the twelve TIN participants was masked by the scanner noise, based on self-report. All TIN participants reported perception of their tinnitus sound after the scan.

### Data Preprocessing

All subject data were preprocessed using statistical parametric software (SPM8, Wellcome Trust Centre for Neuroimaging, http://www.fil.ion.ucl.ac.uk/spm/software/spm8/) in a five-step procedure: slice-time correction, realignment, coregistration, normalization, and smoothing. Scanning was interleaved and ascending; slice-time correction was performed accordingly. Functional images were realigned to the mean fMRI image using a 6-parameter rigid-body transformation to correct for subject head motion. Coregistration was performed in two steps. First, a 12-parameter affine transformation was used to align the TSE image to the mean fMRI image. Then, the MPRAGE image was aligned to the TSE image. Next, this MPRAGE image was normalized to an MNI template using a nonlinear warp transformation, which was then also used on the realigned fMRI images. The normalized, realigned fMRI images were spatially smoothed using a Gaussian kernel of 10 × 10 × 10 mm^3^. Finally, the MPRAGE image was segmented to create images containing solely gray matter, white matter, and cerebrospinal fluid for later use.

### Data Analysis

#### (a): Independent Component Analysis

Group ICA was performed using GIFT software [36] for MATLAB (Mathworks, Inc) and the preprocessed fMRI images for each subject. The analysis was performed separately on each group (TIN, HL, and NH) using 30 components. The Infomax algorithm was used, and the group ICA analysis used was *Icasso* [37]. Components were spatially sorted using a mask for the network being examined. For the auditory RSN, a mask of bilateral Brodmann areas 41 and 42 was created using the WFU Pickatlas [38,39]. For the DMN, a template provided with the GIFT software was used. For the DAN, the mask consisted of five 10 mm-diameter spheres in the left and right frontal eye fields, right ventral intraparietal sulcus, and left and right posterior intraparietal sulci. The coordinates were chosen to be the same as those used by Burton et al. [23] and are listed in Table 2.

**Table 2:** MNI coordinates of seed and mask regions for each analysis.

**Analysis**	**Network**	**Seed/mask Region**	**Coordinates (MNI)**		
			**x**	**y**	**z**
**Group ICA**	**DAN**	left frontal eye field	-25	-11	54
		right frontal eye field	27	-11	54
		right ventral intraparietal sulcus	30	-83	13
		left posterior intraparietal sulcus	-23	-70	46
		right posterior intraparietal sulcus	26	-62	53
**Seeding**	**Auditory**	left primary auditory cortex	55	-22	9
		right primary auditory cortex	-41	-27	6
	**DMN**	medial prefrontal cortex	8	59	19
		posterior cingulate cortex	-2	-50	25
	**DAN_1**	left posterior intraparietal sulcus	-23	-70	46
		right posterior intraparietal sulcus	26	-62	53
	**DAN_2**	left frontal eye field	-25	-11	54
		right frontal eye field	27	-11	54

#### (b): Seeding Analysis

Seeding was performed using the Functional Connectivity Toolbox (Conn) [40] for MATLAB. Prior to seeding, all fMRI data was filtered using a band-pass filter from 0.008 to 0.08 kHz within the Conn toolbox. White matter and cerebrospinal fluid were segmented from the MPRAGE file of each subject individually in SPM8. This data and the motion regressors created during the realignment step of preprocessing were loaded into the toolbox for each subject and were regressed out. Seed-to-voxel analysis was performed to analyze the auditory RSN, the DMN, and the DAN using seeds 10 mm in diameter. For the auditory RSN, seeds were located in the bilateral primary auditory cortices. For the DMN, they were located in the medial prefrontal cortex and the posterior cingulate cortex. For the DAN, two groups of seeds were examined separately. The first group contained spheres representing the left and right posterior intraparietal sulci (referred to as DAN_1), while the second included the left and right frontal eye fields (DAN_2) [23]. Information about the seed regions is listed in Table 2. This analysis determined the connectivity of a specified seed region with the whole brain. The connectivity of the two seed regions in each network were averaged together. Because of the need to average across seed regions, we elected to split the DAN into two seed groups; we did not want to combine together the connectivity of four seed regions and thereby conceal regions of significance. After the toolbox had calculated the connectivity for each individual subject, group averages were computed.

Correlation maps of seed regions were made for each network for each group and were displayed at a threshold of p<0.05 FDR corrected. Across-group comparisons were obtained by employing one-way ANOVA in the Conn toolbox. Results were then exported to SPM8 for display and to conduct post-hoc t-tests. For the one-way ANOVA, a threshold of p<0.001 uncorrected was used in order to avoid the inadvertent exclusion of critical nodes by too conservative a threshold. For the post-hoc two-sample t-tests, a more conservative threshold was used: after whole brain analysis at p<0.001 uncorrected threshold, clusters that were significant at p <0.05 FDR either at voxel or cluster level were selected, with cluster extent set at 25 voxels.

MarsBar [41] and the REX [42] toolbox were used following the t-tests to examine the beta values at significant regions for each individual subject. MarsBar was used to create ROI masks (spheres of 10 mm diameter) centered at the local maxima of each cluster found to be significant during the post-hoc t-tests. REX toolbox was used to extract the mean of the single-subject beta values across each ROI mask. The beta values were not scaled and are depicted as scatter plots of beta values vs. subject group; these plots are included as Figure S1.

## Results

### Independent Component Analysis

**Figure 2:**
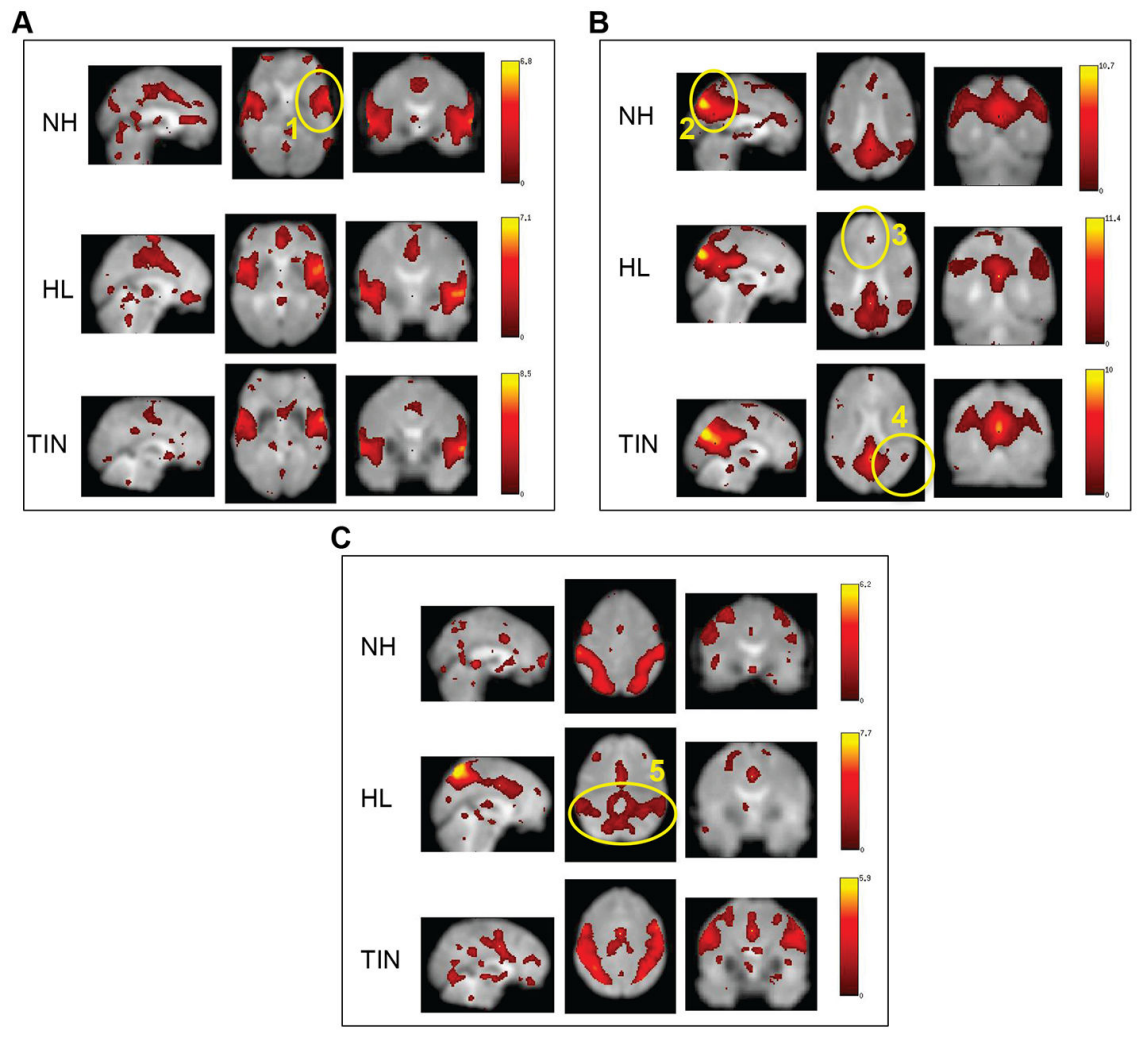
GIFT analysis results. **A**) GIFT results of the auditory network. Visually, there were no large differences between groups in the primary auditory cortices (1). **B**) GIFT results of the default mode network (DMN). Visually, the TIN and HL groups exhibited reduced response in the medial prefrontal cortex (3), whereas the inferior parietal lobules (4) appear to be least functionally coupled in the TIN group. The other main node of the network (posterior cingulate, 2) does not seem to be different in its connectivity across the groups. **C**) GIFT results of the dorsal attention network (DAN). Group differences were not apparent. However, there is a somewhat decreased response in the intraparietal sulci (5) in the HL group.

The auditory RSN, DMN, and DAN were all located within 30 components for each group using spatial sort, with each network visually matching the patterns in fluctuations similar to those found by other studies [2,4]. Visual inspection of the component related to auditory processing suggested no major differences between groups, including in the primary auditory cortices, shown in Figure 2A. In the DMN, the TIN and HL groups showed reduced connectivity in the medial prefrontal cortex as compared to the NH. Further, the TIN group exhibited reduced correlations in bilateral inferior parietal lobules. This is shown in Figure 2B. As depicted in Figure 2C, there were no large differences between groups for the DAN components, although there is a small decrease in the connectivity of the intraparietal sulci in the HL group when qualitatively compared to the other groups.

No statistical analyses of the independent component analysis have been included here. This is because a separate analysis was conducted on each group; any differences found between groups could be artifactual due to the individual group ICA decompositions performed and not related to intrinsic differences. We therefore conducted a seeding analysis to provide a more accurate estimation of across-group differences.

### Seeding Analysis

#### (a): Auditory Resting State Network

**Figure 3:**
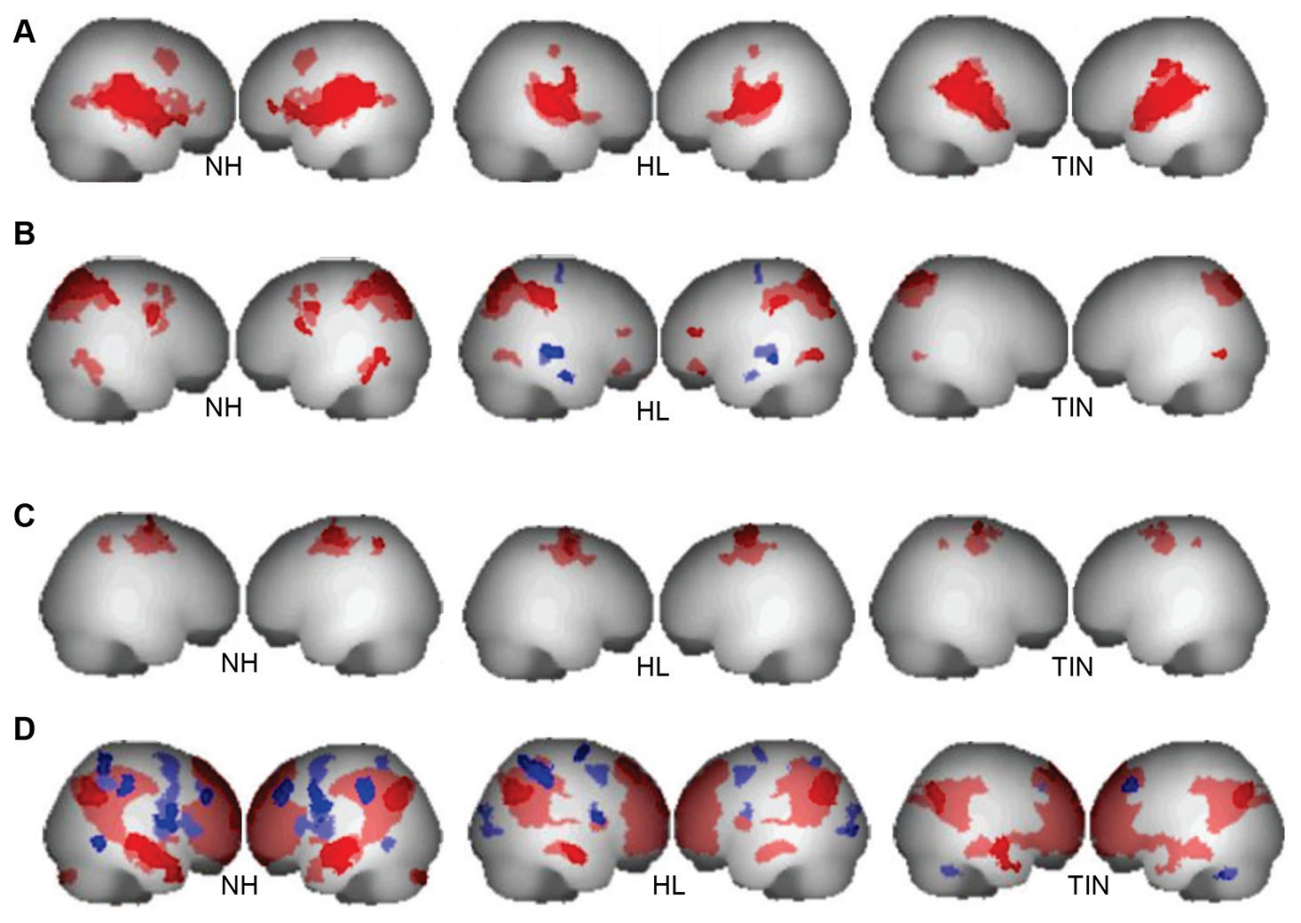
Correlation maps of seed regions for each network. Correlations shown are p<0.05 FDR corrected significant at the voxel and/or cluster level. Red represents positive correlations with seeds, while blue shows negative correlations. **A**) Correlations with the seed regions in the auditory resting state network. Visually, there does not appear to be strong differences across groups, though the HL group shows slightly reduced connectivity when compared to the other two groups. **B**) Correlations with the first pair of seeds in the dorsal attention network (DAN_1). There seems to be reduced correlations in the TIN group compared to the other two groups, as well as negative correlations that are exclusive to the HL group. **C**) Correlations with the second pair of seeds in the dorsal attention network (DAN_2). Few differences can be noted across groups. **D**) Correlations with the seed regions in the default mode network (DMN). Reduced connectivity (especially negative correlations) from the DMN seeds to other brain regions is seen in the TIN group when compared to the other two groups.

**Figure 4:**
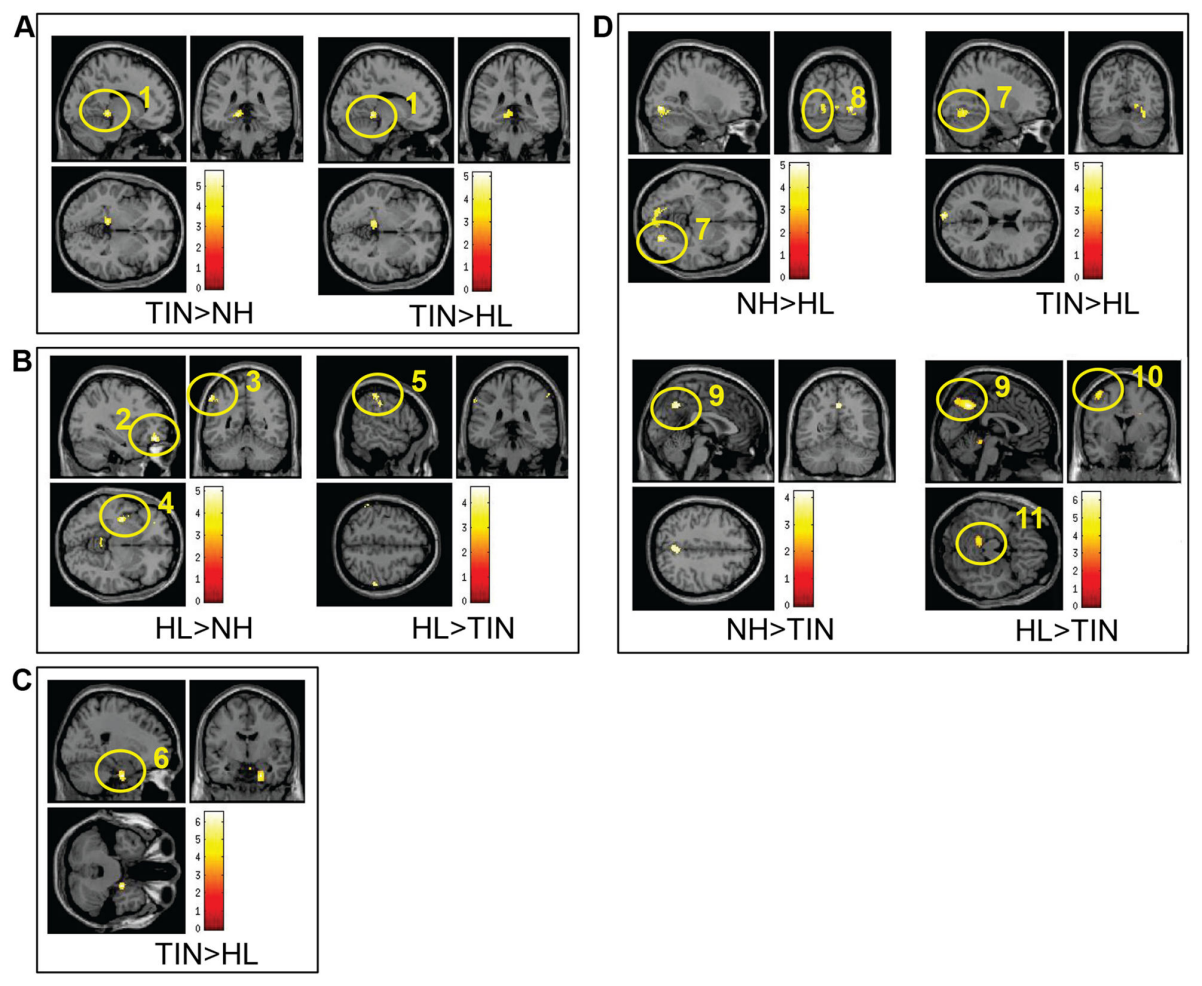
Post-hoc t-tests of seeding analysis results. **A**) The TIN>NH and TIN>HL comparisons for the auditory network. In the TIN>NH contrast, a significant difference was found in the left lingual gyrus into the left parahippocampus (1). There was also a trend for this same result in the TIN>HL comparison (also labeled as 1), but it did not reach p<0.05 FDR corrected significance (it was significant at p<0.1 FDR corrected). **B**) The HL>NH and HL>TIN comparisons for the first seed pair of the dorsal attention network. Significant differences were found in the left middle orbital gyrus (2), left inferior parietal lobule (3) and left insula (4) in the HL>NH contrast, and in the right supramarginal gyrus (5) in the HL>TIN comparison. **C**) The TIN>HL contrast for the second dorsal attention seed pair. The only significant difference seen occurred in the right parahippocampal gyrus (6). **D**) The NH>HL, HL>TIN, TIN>HL, and NH>TIN contrasts for the default mode network. In the NH>HL and NH>TIN contrast, a significant difference was found in the right fusiform gyrus (7). The bilateral lingual gyri (8) were also significant in the NH>HL contrast. The precuneus (9) is significant in both the NH>TIN and HL>TIN contrasts. The left precentral gyrus (10) and the left cerebellum (11) were significant in the HL>TIN contrast as well.

Visual inspection of the correlation maps did not reveal large differences across groups. However, connectivity appeared to be reduced in the HL group (see Figure 3A). ANOVA analysis revealed an effect of group in the left lingual gyrus and the left parahippocampus for the auditory RSN. We conducted post-hoc two-sample t-tests to identify the source of the group effect (see Figure 4A and Table 3). For the TIN>NH contrast, significant connectivity differences were seen in the left lingual gyrus and left parahippocampus. Although it did not reach p<0.05 FDR corrected significance, there was a trend toward significance in the left lingual gyrus for the TIN>HL contrast as well (the region reached significance at p<0.1 FDR corrected). A scatter plot of the beta values in left lingual gyrus confirmed this result and further suggests that the TIN>NH contrast showed stronger results because the beta values were more negative in the NH group (see Figure S1A). No other contrasts revealed significant results.

**Table 3:** Local maxima for between-group contrasts of the auditory resting state network (seeding analysis).

**Contrast**	**Coordinates (MNI)**					**Z-Score**	**Cluster Size**	**Gyrus**
	**x**		**y**		**z**			
**NH>HL**	None							
**HL>NH**	None							
**HL>TIN**	None							
**TIN>HL**	None							
**NH>TIN**	None							
**TIN>NH**	-12	-36		-2		4.82*	113	Left lingual gyrus
	-24	-42		-4		3.34*		Left parahippocampus

Regions are reported in Montreal Neurological Institute (MNI) coordinates. All reported clusters are p≤0.050 FDR corrected for multiple comparisons at the voxel (indicated by # next to the Z-score) or cluster-level (indicated by *). Cluster extent=25 voxels.

#### (b): Dorsal Attention Network

Connectivity maps showed that some brain regions, which were negatively correlated with the DAN_1 seeds (bilateral posterior intraparietal sulci) in the HL group, were not present in the results of the NH or TIN groups. For the TIN group, there were fewer significant correlations overall (Figure 3B). The maps did not visually show connectivity differences for the DAN_2 seeds (bilateral frontal eye fields) across groups (Figure 3C). For the DAN_1 seeds, an effect of group was seen in the left cerebellum, left middle orbital gyrus, and the left rolandic operculum. The results of the post-hoc t-tests following the ANOVA are shown in Figure 4B and Table 4. No suprathreshold connectivity patterns were found in gray matter for the NH>HL, NH>TIN, TIN>HL, and TIN>NH comparisons. In the HL>TIN condition, differences in brain connectivity were seen in the right supramarginal gyrus. This was confirmed by the scatter plot of the beta values from this region (Figure S1B). Lastly, in the HL>NH connectivity contrast, suprathreshold voxels were found in the left middle orbital gyrus, the left inferior parietal lobule and the left insula lobe, and the scatter plots confirmed the results (Figure S1B).

**Table 4:** Local maxima for between-group contrasts of the dorsal attention network using bilateral intraparietal sulci as seeds (DAN_1) (seeding analysis).

**Contrast**	**Coordinates (MNI)**			**Z-Score**	**Cluster Size**	**Gyrus**
	**x**	**y**	**z**			
**NH>HL**	None					
**HL>NH**	-32	44	-14	4.45*	130	left middle orbital
	-58	-50	46	4.44*	120	left inferior parietal
	-48	-50	44	3.46*		left inferior parietal
	-38	-10	-6	4.28*	93	left insula
	-40	-2	-8	3.53*		left insula
**HL>TIN**	58	-32	50	3.84*	116	right supramarginal
	58	-24	40	3.65*		right supramarginal
**TIN>HL**	None					
**NH>TIN**	None					
**TIN>NH**	None					

Regions are reported in Montreal Neurological Institute (MNI) coordinates. All reported clusters are p≤0.050 FDR corrected for multiple comparisons at the voxel (indicated by # next to the Z-score) or cluster-level (indicated by *). Cluster extent=25 voxels.

In the DAN_2 seeds, an effect of group was seen in the right parahippocampus. Post-hoc t-tests revealed a significant difference in the right parahippocampus in the TIN>HL contrast. No other contrast showed above threshold voxels. These results are shown in Figure 4C and Table 5 and are confirmed by the scatter plot in Figure S1C.

**Table 5:** Local maxima for between-group contrasts of the dorsal attention network using bilateral frontal eye fields as seeds (DAN_2) (seeding analysis).

**Contrast**	**Coordinates (MNI)**					**Z-Score**	**Cluster Size**	**Gyrus**
	**x**		**y**		**z**			
**NH>HL**	None							
**HL>NH**	None							
**HL>TIN**	None							
**TIN>HL**	22	-6		-34		5.29#*	144	right parahippocampus
	28	-12		-32		4.22*		right parahippocampus
**NH>TIN**	None							
**TIN>NH**	None							

Regions are reported in Montreal Neurological Institute (MNI) coordinates. All reported clusters are p≤0.050 FDR corrected for multiple comparisons at the voxel (indicated by # next to the Z-score) or cluster-level (indicated by *). Cluster extent=25 voxels.

#### (c): Default Mode Network

Visually, the connectivity maps were different across groups. There was a large decrease in correlations with the seed regions in the TIN group when compared to the two controls. In particular, there were fewer regions negatively correlated with the seed regions (see Figure 3D). An effect of group was found for the DMN after seeding analysis in the left precuneus, the left cuneus, the right fusiform gyrus, and the left and right lingual gyri. The results of post-hoc t-tests are shown in Figure 4D and Table 6. No differences were seen in the default mode connectivity patterns for the HL>NH and TIN>NH contrasts. In the NH>HL contrast, connectivity differences were seen in right fusiform gyrus and bilateral lingual gyri. The NH>TIN contrast showed a significant result only in the right precuneus. Comparison of the HL and TIN groups revealed significant results in both directions. For the HL>TIN contrast, left precuneus, left precentral gyrus, and left cerebellum showed significant connectivity differences. In the TIN>HL comparison, the differences were in the right fusiform and right lingual gyri. The right fusiform/lingual gyrus scatter plot in Figure S1D confirmed the connectivity differences seen in the TIN>HL and NH>HL contrasts. Figure S1D also shows the scatter plots for the bilateral precuneus, left cerebellum, and left precentral gyrus, which corroborated the results seen in the NH>TIN and HL>TIN contrasts.

**Table 6:** Local maxima for between-group contrasts of the default mode network (seeding analysis).

**Contrast**	**Coordinates (MNI)**				**Z-Score**	**Cluster Size**	**Gyrus**
	**x**	**y**		**z**			
**NH>HL**	28	-78	-6		4.40*	171	right fusiform
	28	-64	-10		3.65*		right fusiform
	28	-72	-12		3.62*		right fusiform
	-16	-78	-2		4.37*	217	left lingual
	6	-80	-4		3.89*		right lingual
	-6	-84	-8		3.61*		left lingual
**HL>NH**	None						
**HL>TIN**	0	-56		40	5.26#*	879	left precuneus
	-10	-58		46	4.58*		left precuneus
	-6	-68		52	3.89*		left precuneus
	-40	0		60	4.61*	137	left precentral
	-12	-42		-18	4.20*	163	left cerebellum
	0	-40		-12	3.65*		cerebellar vermis
**TIN>HL**	26	-66		-10	3.94*	163	right fusiform
	18	-66		-2	3.86*		right lingual
**NH>TIN**	2	-58	42		3.80*	106	right precuneus
**TIN>NH**	None						

Regions are reported in Montreal Neurological Institute (MNI) coordinates. All reported clusters are p≤0.050 FDR corrected for multiple comparisons at the voxel (indicated by # next to the Z-score) or cluster-level (indicated by *). Cluster extent=25 voxels.

## Discussion

We investigated resting state functional connectivity in adults with hearing loss and non-bothersome tinnitus and compared the results to two groups without tinnitus – those with matched hearing loss and those with normal hearing. We focused on three resting state networks: the auditory network (seeds in the bilateral primary auditory cortices), the DMN (with seeds in the posterior cingulate and medial prefrontal cortex), and the DAN. The DAN was probed using two sets of seeds, DAN_1 (the bilateral posterior intraparietal sulci) and DAN_2 (bilateral frontal eye fields). Henceforth, DAN_1 and DAN_2 will be used to refer to the specific seeds; DAN will be used to refer to the dorsal attention network in general. Group ICA analysis was primarily used to provide an unbiased, data-driven visualization of each network. Our ability to compare the networks between groups was inhibited by the fact that three separate ICA decompositions were performed (one each for the NH, HL and TIN groups). Thus, differences between the groups could be attributed to the decompositions and not to tinnitus or hearing loss. Therefore, we chose to concentrate on a seeding analysis to determine group differences and for the remainder of this section, we discuss results of the seeding analysis only. Because we were primarily interested in the comparison of the TIN group with the two control groups, we do not discuss the differences between the NH and HL groups; however, we report them in the tables. Seeding analysis revealed increased connectivity between the parahippocampus and the DAN (via DAN_2 seeds) and auditory RSN in subjects with TIN as compared to HL and NH controls, respectively. Results also revealed reduced connectivity between the DAN_1 seeds and right supramarginal gyrus, a region also associated with attention, in the TIN group when compared to HL controls. Seeding analysis further revealed decreased connectivity between the DMN seed regions and the precuneus, a primary hub of the DMN [2,4,5].

### Auditory Resting State Network

Animal and human studies have demonstrated that extra-auditory regions, including regions in the limbic system [33,34], somatosensory system [35], and attention processing system [22,30] have altered response patterns in individuals with tinnitus compared to healthy controls. We had hypothesized that such alterations may be reflected in the functional connectivity patterns from the auditory RSN to these regions in the tinnitus population. Contrary to our hypothesis, we saw few changes in the auditory RSN when comparing TIN to the control groups. However, the left parahippocampus, part of the limbic system, was found to be more strongly connected to the network in the TIN group when compared to NH controls. This trend was also present when the TIN group was compared to the HL controls, although it did not reach significance. These results strongly suggest that chronic tinnitus, and not the accompanying hearing loss, is responsible for the increased connectivity with the parahippocampal gyrus.

The influence of the limbic system has been noted in other resting state studies of tinnitus [24–26] with contradictory findings. In one such study [26], connectivity graph analysis of the auditory RSN showed increased connectivity with the brainstem, cerebellum, right basal ganglia, and parahippocampal areas in patients with varying tinnitus severity when compared to NH controls (THI scores of those subjects ranged from 16 to 84, with most scores in the 30s and 40s). Our finding of increased connectivity between the auditory RSN and the left parahippocampus partially supports these results. An initial study by Kim et al. [24] also found increased connectivity between the auditory RSN and the limbic system, specifically in the left amygdala and dorsomedial prefrontal cortex. In our study, we did not see increased connectivity to the amygdala, but the connectivity with the parahippocampal gyrus supports the idea that the limbic system is involved with the auditory RSN in tinnitus. Our results, nevertheless, run contrary to the study by Wineland et al. [27], which found no significant effects when examining patients with nonbothersome tinnitus who had THI scores similar to those in our study (0-24 in [27], 0-22 in this study). It is possible that this difference is due to the variable hearing loss in the population examined by Wineland et al. [27].

### Dorsal Attention Network

Mantini et al. [4] and others [2,43] have shown that the DAN exhibits reduced response at rest in brain regions that are functionally responsive during a task. In our previous task-based fMRI study [22], we saw decreased response of some nodes and elevated response of other nodes in the TIN group relative to NH controls, but the HL group showed a uniformly elevated activation pattern compared to the NH controls. Accordingly, we expected the DAN to show decreased coherence in TIN subjects compared to either of the control groups because the act of suppressing their tinnitus would result in a differential response pattern across the network nodes. Our results suggest a complicated relationship between tinnitus, hearing loss, and attention, echoing the Husain et al. [22] study. The right supramarginal gyrus was significantly correlated with the DAN_1 seeds in the HL>TIN comparison, which suggests that the perception of the phantom sound, rather than hearing loss, is modulating this connection with an attention processing node. We also observed increased connectivity between the right parahippocampus and the DAN_2 seeds in the TIN>HL contrast. The increase in correlation could be a compensatory attempt to manage the phantom stimulus, delegating that process to non-attention processing regions, such those of the limbic system [33,34]. It is important to note that other studies [23,27] that examined the DAN in tinnitus did not find any connectivity differences in the network. As mentioned previously, this difference in results could be due to methodological differences or variable hearing loss in the TIN groups in the other studies.

The act of attending to or ignoring the tinnitus stimulus could lead to the results seen in this study. Given that a third of the participants reported that their tinnitus was masked by the scanner noise, something other studies have not discussed, it is possible that a mixture of the two scenarios is present here. Future imaging studies that incorporate both attention-related and resting tasks in an interleaved fashion are necessary to identify the precise change in state during rest-to-task transition at the individual subject level.

### Default Mode Network

Differences in the DMN across the groups followed similar patterns as those in the DAN. The DMN has been shown to exhibit decreased response when subjects move into a task-based mode, which engages the DAN, and shows increased response when the DAN is not activated [2]. This push-pull relationship between the two networks led to the hypothesis that in the TIN group, the DMN would be less engaged because the subjects are in a more task-based state due to perception of the internal noise. Our results are in agreement with this hypothesis. When compared to both HL and NH controls, the TIN group showed reduced connectivity between the seed regions and bilateral precuneus. The precuneus is one of the main nodes of the DMN [2,4,5], and the decrease in connectivity with the seed regions suggests that the network is less coherent in patients with tinnitus. The HL>TIN results further demonstrated that the reduced connectivity with the precuneus was not due to hearing loss and may therefore be attributed to the phantom sound. In fact, the result was more strongly significant when compared to HL than NH controls, possibly due to increased variability in the NH group (see Figure S1D). The precuneus has been implicated in consciousness and self-reflection [44]. In resting state studies, precuneus deactivations have been shown to occur in many different disorders, including Alzheimer’s disease and schizophrenia [44]. It is not surprising that this area is implicated in tinnitus as well. However, differences in precuneus connectivity have not been noted in other resting state studies of tinnitus. In several studies [24–26], this is likely because the focus was only on the auditory network. In the Burton et al. [27] and Wineland et al. [28] studies, methodological differences and variable hearing loss in the tinnitus groups may be responsible for this variation in findings.

The other significant result in the DMN was the increased connectivity between the seed regions and the right fusiform and lingual gyri in the TIN compared to the HL group. These loci adjoin the parahippocampus, which is an area of significance in the auditory and attention networks.

### Possible Confounds

It is important to note that the presence of scanner noise makes it challenging to draw conclusions about the behavior of resting state networks in tinnitus. Though we attempted to minimize the influence of scanner noise with earplugs and headphones, the scanner sound was audible during the continuous data acquisition. All three subject groups were passively listening to or trying to ignore an auditory stimulus; the TIN group was not unique in this regard. It is possible that a true resting state is not actually achieved in an fMRI study as a consequence of this. In fact, scanner noise has been shown to disrupt the DMN in normal hearing individuals [45]. It is important to keep this observation in mind when comparing resting state fMRI studies to resting state studies employing other quieter imaging techniques such as EEG [46,47] or MEG [48–51]. Further complicating the issue, four out of our twelve subjects reported their tinnitus sound was masked by the scanner noise, a question that has not been asked in previous resting state tinnitus studies. Such masking may cause these participants of the TIN group to move into a tinnitus-free state temporarily, and thus their intrinsic networks may resemble those of the HL group. However, the small size of the subject group who experienced masking makes it difficult to compare their RSNs with those of the larger groups; this comparison was therefore not made in this study.

The groups in our study were both age and gender-matched to the best of our ability, though there is some slight variation in both categories between groups (the HL group tended to be older and contained more females). Our first priority was to match the extent of hearing loss in the HL and TIN groups, then to match all participants across the three groups in terms of age and finally to match on the basis of gender. It has been shown in several studies [52–54] that aging can affect rs-fMRI; it is therefore possible that some of the effects of tinnitus or hearing loss seen in these groups may be a result of aging. However in our groups, a one-way ANOVA did not reveal a main effect of age (F[2, 37] =1.07, p=0.3527). This, coupled with the small group sizes, makes it infeasible to address the effect of aging on the results. A larger study designed to assess the impact of aging within the context of hearing loss or tinnitus should be performed. Gender can also have an impact on resting state connectivity [54]. Though a one-way ANOVA on this variable did not reveal a main effect of gender (F[2, 37]=1.76, p=0.1866), the gender imbalance between subject groups is something that must be considered when assessing the results of this study. Again, the small subject groups make it difficult to assess what the effect of gender may be and a larger study would be more adept to answer this question.

## Conclusion

The main finding of the study was increased functional connectivity between limbic regions and the auditory and the dorsal attention resting state networks in TIN participants, compared to the NH and HL control groups, respectively. The coupling of the limbic regions with these networks is indicative of the relationship between the limbic system and tinnitus frequently discussed in the literature and agrees with previous studies of resting state connectivity in tinnitus [24–26]. However, our results do not agree with a study specifically examining nonbothersome tinnitus [27], possibly due to the fact that the previous study examined patients with variable hearing loss. In addition, in the dorsal attention network, TIN patients showed decreased correlations with brain regions associated with attention, suggesting a role of the attention network in diminishing the impact of the chronic internal sound. The precuneus was found to have significantly reduced connectivity with other nodes of the default mode network in tinnitus patients relative to both control groups, suggesting that the tinnitus percept is shifting patients away from a true resting state. This may be partially responsible for the mental fatigue that is often experienced by tinnitus patients; even when others are in a resting state, they are not. Therapies for tinnitus that address the increased connectivity of limbic regions with auditory and attention resting state networks and a decreased coherence of the default mode network may result in diminution of tinnitus-related distress.

## Supporting Information

Figure S1 **Scatter plots of individual subject beta values in regions of significance.** **A**) The scatter plot for areas of significant differences in the auditory network (left lingual gyrus and left parahippocampus). The plot shows less variability in the HL group compared to the other two groups and partially explains why this region did not reach significance in the TIN>NH condition.
**B**) The scatter plots for the areas of significant differences in the first pair of seeds representing the dorsal attention network (DAN_1). The plots illustrate the significant results in the HL>NH contrast in the left middle orbital gyrus, the left insula lobe, and the left inferior parietal lobule. They also demonstrate that the result in the right supramarginal gyrus in the HL>TIN contrast may be due to reduced variability in the TIN group in addition to the lower beta values.
**C**) The scatter plot for the significant difference in the post-hoc t-tests for the second pair of seeds for the dorsal attention network (DAN_2). The TIN>HL contrast showed a significant difference only in the right parahippocampus, which is explained by the scatter plot. However, the TIN>NH did not reach significance possibly due to the greater variability.
**D**) The scatter plots for the areas of significant differences in the default mode network (DMN). The plots support the results of the two-sample t-tests. The right fusiform and right lingual gyri (combined into one plot due to their close proximity) was significant in NH>HL and TIN>HL comparisons, whereas the bilateral precuneus was significant in the NH>TIN and HL>TIN contrasts. The left precentral gyrus and the left cerebellum were both significant in the HL>TIN contrasts. In the left precentral gyrus, the NH and TIN subjects display similar beta values. In the left cerebellum, the high beta values of two of the NH participants may underlie the non-significance of the results in the HL>NH contrast.
(TIF)
