# Supplementary figures and images for: Default Mode, Dorsal Attention and Auditory Resting State Networks Exhibit Differential Functional Connectivity in Tinnitus and Hearing Loss
Source: PLoS One. 2013 Oct 2;8(10):e76488. doi: 10.1371/journal.pone.0076488 (PMC3788711; doi:10.1371/journal.pone.0076488)

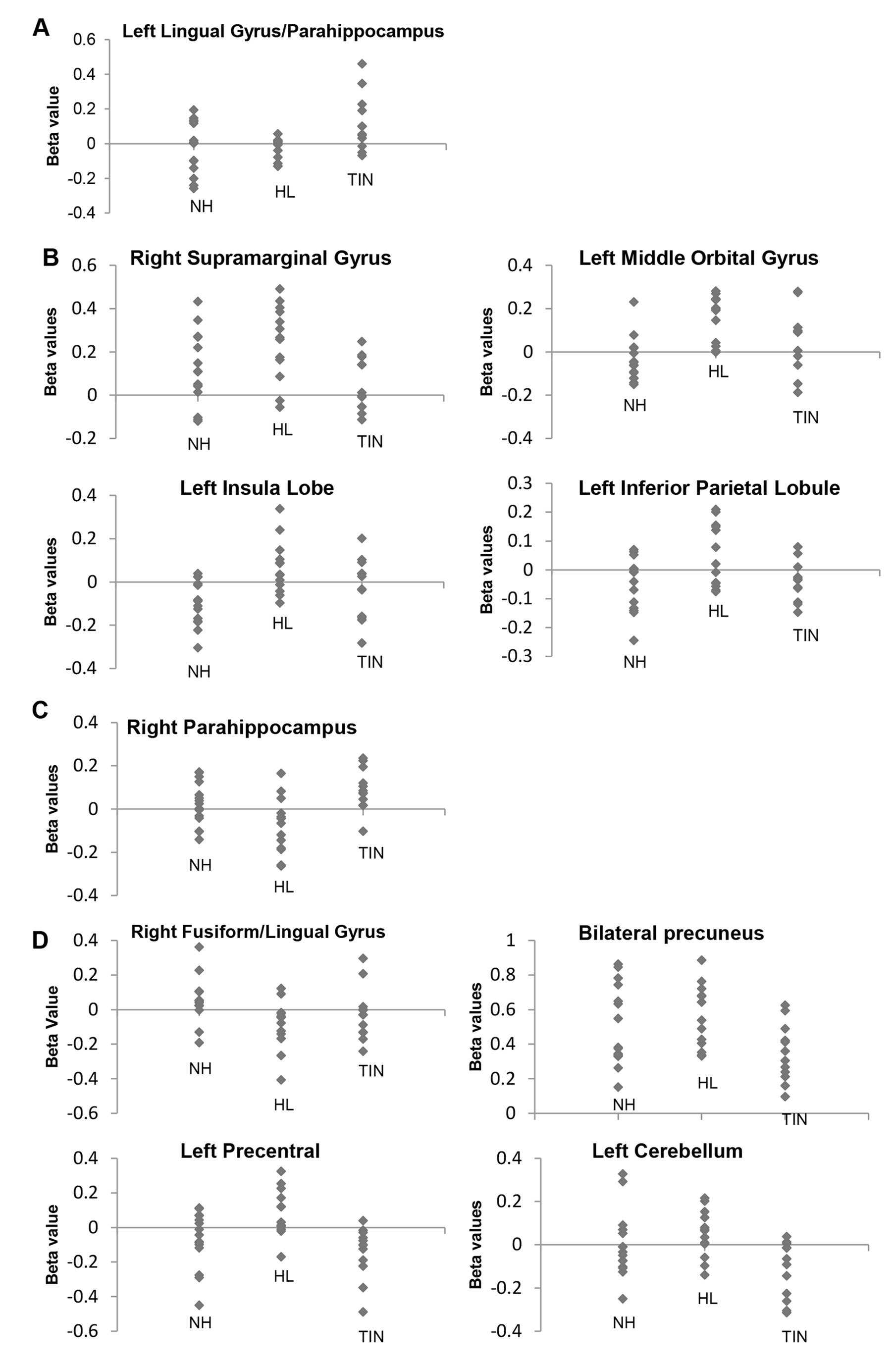

Supplement: Figure S1 — Scatter plots of individual subject beta values in regions of significance. A) The scatter plot for areas of significant differences in the auditory network (left lingual gyrus and left parahippocampus). The plot shows less variability in the HL group compared to the other two groups and partially explains why this region did not reach significance in the TIN>NH condition. B) The scatter plots for the areas of significant differences in the first pair of seeds representing the dorsal attention network (DAN_1). The plots illustrate the significant results in the HL>NH contrast in the left middle orbital gyrus, the left insula lobe, and the left inferior parietal lobule. They also demonstrate that the result in the right supramarginal gyrus in the HL>TIN contrast may be due to reduced variability in the TIN group in addition to the lower beta values. C) The scatter plot for the significant difference in the post-hoc t-tests for the second pair of seeds for the dorsal attention network (DAN_2). The TIN>HL contrast showed a significant difference only in the right parahippocampus, which is explained by the scatter plot. However, the TIN>NH did not reach significance possibly due to the greater variability. D) The scatter plots for the areas of significant differences in the default mode network (DMN). The plots support the results of the two-sample t-tests. The right fusiform and right lingual gyri (combined into one plot due to their close proximity) was significant in NH>HL and TIN>HL comparisons, whereas the bilateral precuneus was significant in the NH>TIN and HL>TIN contrasts. The left precentral gyrus and the left cerebellum were both significant in the HL>TIN contrasts. In the left precentral gyrus, the NH and TIN subjects display similar beta values. In the left cerebellum, the high beta values of two of the NH participants may underlie the non-significance of the results in the HL>NH contrast. (TIF) [file pone.0076488.s001.tif]
